# Analysis of the Serum Lipid Profile in Polypoidal Choroidal Vasculopathy

**DOI:** 10.1038/srep38342

**Published:** 2016-12-02

**Authors:** Miaoling Li, Xiongze Zhang, Nanying Liao, Baikang Ye, Yuting Peng, Yuying Ji, Feng Wen

**Affiliations:** 1 State Key Laboratory of Ophthalmology, Zhongshan Ophthalmic Center, Sun Yat-sen University, Guangzhou, China

## Abstract

Polypoidal choroidal vasculopathy (PCV), the predominant subtype of neovascular age-related macular degeneration in the Asian population, is associated with genetic polymorphism of lipid metabolism. In this study, we performed the untargeted lipidomics approach of ultra-performance liquid chromatography coupled with mass spectrometry (UPLC-MS) to reveal the potential discriminating lipid profile of PCV patients in serum (21 PCV patients and 19 age-matched controls). Unsupervised principal component, supervised orthogonal partial least squares analysis, correlation analysis, and heatmap analysis were performed with the data obtained by UPLC-MS. Forty–one discriminating metabolites were identified. Receiver operating characteristic curve analysis, pathway analysis and functional analysis were performed subsequently, and platelet-activating factor (PAF) was further selected as the key indicator of the distinct lipid metabolism in PCV patients. Finally, the serum level of PAF was validated by enzyme-linked immunosorbent assay, which is significantly higher in PCV patients compared to controls (65 PCV patients and 63 age-matched controls, p < 0.0001), consistent with the UPLC-MS analysis. Our results suggested that PAF is considered as the major indicator of the distinct lipid metabolism in PCV patients.

Polypoidal choroidal vasculopathy (PCV) is a disease characterized by a branching choroidal network with terminal polypoidal dilatations of the choroidal vessels in the macular and/or peripapillary area, which cause episodic leakage and bleeding under the retinal pigment epithelium (RPE) and neurosensory retina^1^,^2^,^3^. It is the predominant subtype (accounting for 22.3–61.6%) of neovascular age-related macular degeneration (nAMD) in the Asian population^4^. Gene polymorphisms have been intensively studied with PCV patients to reveal the pathogenesis of PCV^5^. Previously, we detected a significant association between the polymorphisms in the *cholesteryl ester transferprotein (CETP)* and PCV^6^, which was confirmed by several subsequent studies^7^,^8^,^9^. Moreover, lipid exudate is a common finding in the retinae of patients with PCV^10^. These results suggest that altered lipid metabolism may be involved in the pathogenesis of PCV.

In this study, we aimed to detect the serum lipid profile of PCV patients compared to controls, using untargeted ultra-performance liquid chromatography coupled with mass spectrometry (UPLC-MS).

## Results

### Baseline characteristics

The patients’ demographics, including age, gender, body mass index, history of hypertension, alcohol consumption, smoking status, and serum levels of triacylglycerol (TG), total cholesterol (TC), high-density lipoprotein-cholesterol (HDL-c), and low-density lipoprotein-cholesterol (LDL-c), are summarized in Table 1. The differences of these characteristics were not significant between patients and controls.

### Multivariate statistical analysis of the lipidomics data

**Figure 1:**
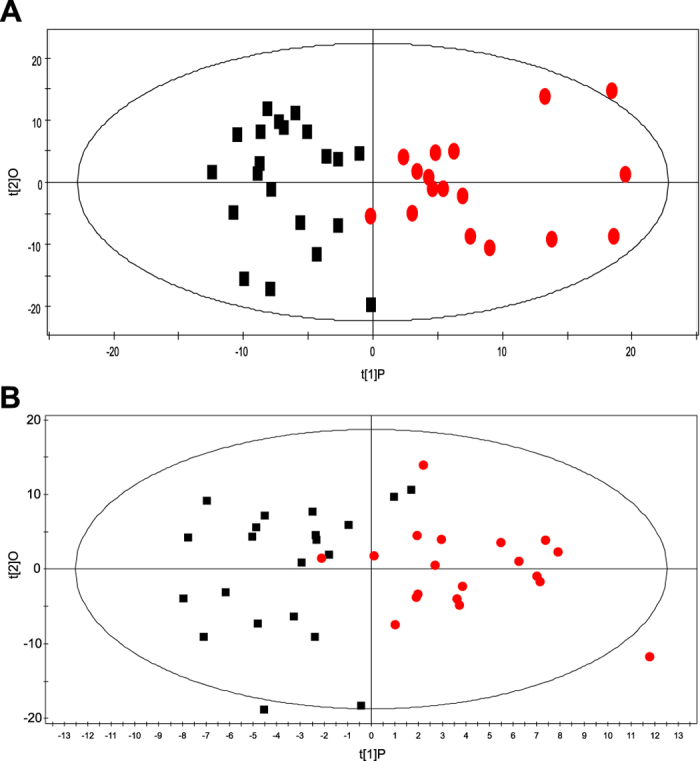
OPLS scores plot of PCV patients and controls. Box (black), PCV patients; circle (red), controls. (**A**) ESI+, R^2^X (cum) = 0.165, R^2^Y (cum) = 0.714, Q^2^ (cum) = 0.148; (**B**) ESI-, R^2^X (cum) = 0.272, R^2^Y (cum) = 0.628, Q^2^ (cum) = −0.013.

**Figure 2:**
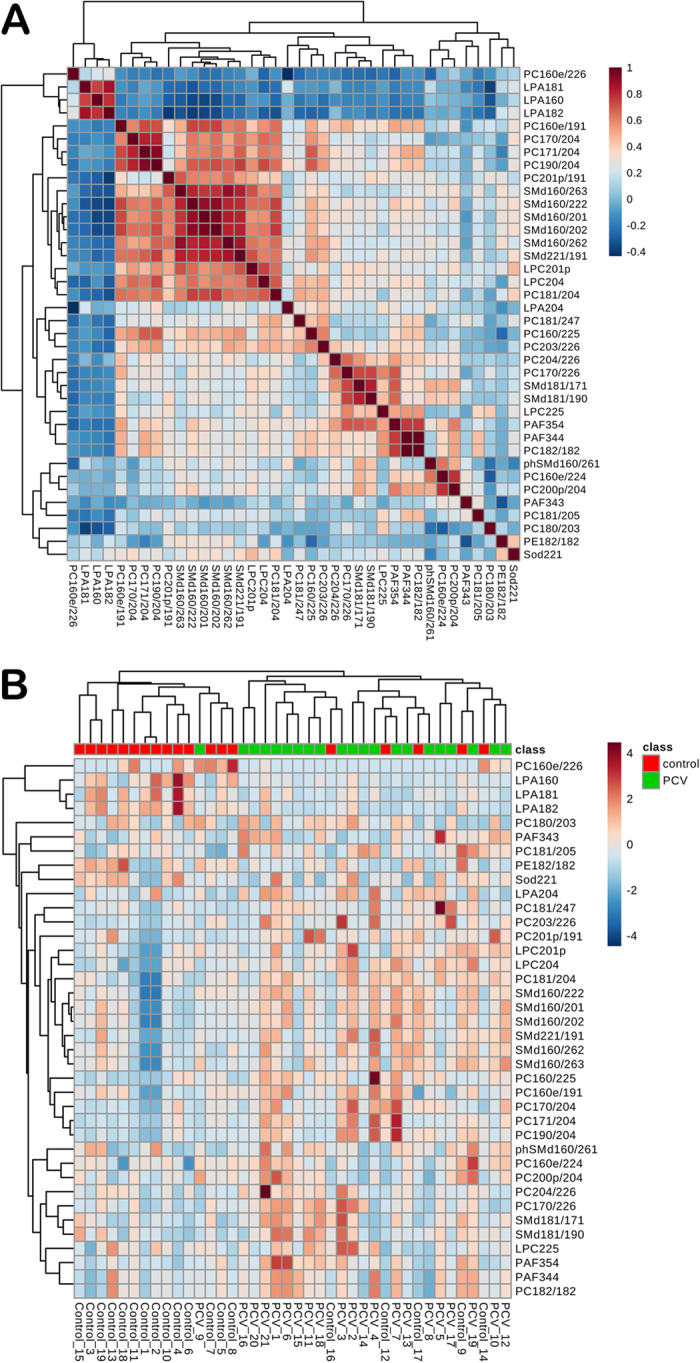
Correlation analysis (**A**) of the differential lipids in PCV patients and controls. Heat map (**B**) for identified lipids in PCV patients and controls. The colour of each section is proportional to the significance of change of lipids (red, up-regulated; blue, down-regulated). Rows: samples; columns: metabolites. Three metabolites-PC (16:1p/20:4), PC (18:0/20:4) and So (d16:0)-are reduced after data filtering by MetaboAnalyst 3.0.

Unsupervised principal component (PCA) and supervised orthogonal partial least squares (OPLS) analysis (Fig. 1) were performed with the data obtained by UPLC-MS. A total of 41 discriminating metabolites were then identified [variable importance for projection (VIP) > 1, p < 0.05, Table 2]. They included 19 phosphatidylcholines (PCs), 8 sphingomyelins (SMs), 4 lysophosphatidic acids (LPAs), 3 platelet-activating factors (PAFs), 3 lyso-PCs (LPCs), 2 sphingosines (Sos), 1 phytosphingosine (phSM) and 1 phosphatidylethanolamine (PE). Their relationships were revealed by correlation analysis (Fig. 2A). To further understand the metabolic differences between PCV patients and controls, the identified lipids data were analysed using clustering heatmap. As shown in Fig. 2B, even though sample clusters overlapped slightly, most samples were clearly grouped into two differentiated clusters, in agreement with the OPLS analysis.

### Receiver Operating Characteristic (ROC) Curve Analysis

Further selection of potential indicator is conducted by ROC analysis. As shown in Fig. S1, LPA (18:2), LPC (20:4), PC (20:1p/19:1), SM (d16:0/22:2), PAF (35:4), PC (16:0/22:5) and PC (18:1/20:4) are the metabolites with area under the ROC curve (AUC) ≥0.8.

### Metabolic Pathway Analysis

**Figure 3:**
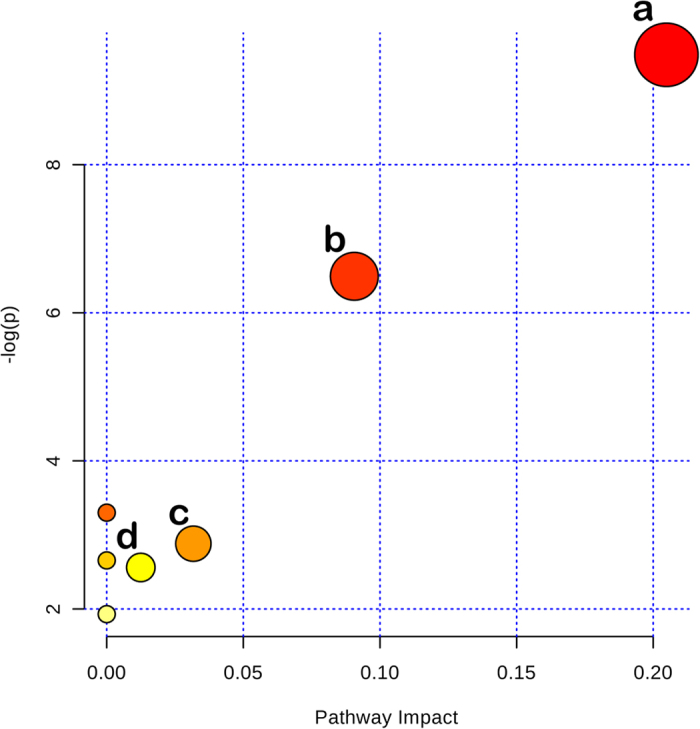
Summary of pathway analysis with MetaboAnalyst 3.0. (**a**) Glycerophospholipid metabolism; (**b**) sphingolipid metabolism; (**c**) ether lipid metabolism; (**d**) glycerolipid metabolism.

Potential target metabolic pathway analysis revealed that the metabolites, identified by multivariate statistical analysis, are responsible for the metabolism of glycerophospholipid, sphingolipid, ether lipid, and glycerolipid (impact-value ≥ 0.00, Fig. 3). The pathways of glycerophospholipid metabolism, ether lipid metabolism and glycerolipid metabolism are closely linked to each other (http://www.kegg.jp/).

### Enzyme-linked Immunosorbent Assay (ELISA) Validation of PAF

**Figure 4:**
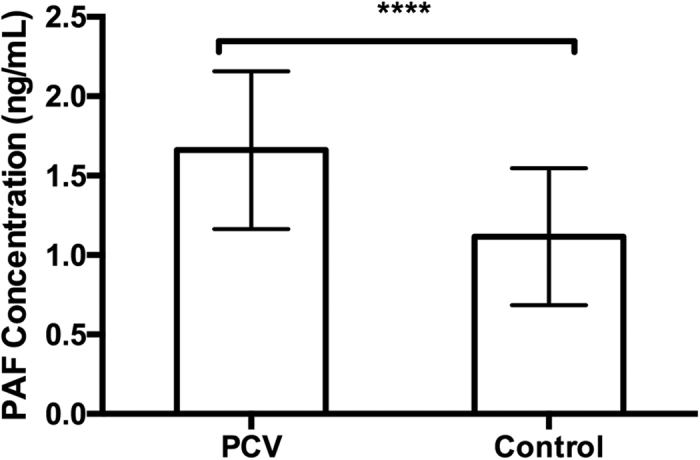
The levels of key indicator PAF determined by ELISA. The difference of PAF levels between PCV patients (n = 65) and controls (n = 63) was assessed using unpaired Student’s t-tests. Two replicates were used. The error bars represent the S.D. of the mean. ****p ≤ 0.0001, compared with controls.

The functions of the identified metabolites, of which both the AUC were ≥0.8 and participating in the key metabolic pathway, were further analysed by public databases, including HMDB (http://www.hmdb.ca), LIPIDMAPS (http://www.lipidmaps.org), METLIN (https://metlin.scripps.edu) and PubMed (http://www.ncbi.nlm.nih.gov/pubmed). Subsequently, PAFs were selected as the key indicators for their important roles in angiogenesis, and were therefore further quantified by ELISA. As shown in Fig. 4, PAF significantly increased in PCV patients (62.43 ± 7.85 years old, 39 male/26 female, 1.66 ± 0.50 ng/mL) compared to controls (65.35 ± 11.28 years old, 24 male/39 female, 1.12 ± 0.43 ng/mL, p < 0.0001), which is consistent with the UPLC-MS analysis (Table 2).

## Discussion

In this study, we screened the potential distinct lipid species of PCV patients compared with controls using untargeted UPLC-MS. Minimization of the potential influence from systemic confounding factors was considered. Therefore, strict inclusion and exclusion criteria for patients and controls were applied. First, patients or controls with diabetes or coronary artery disease (CAD) were excluded from our study. Moreover, all control subjects were matched to PCV participants on gender, age and serum level of TC and TG. Lastly, smoking status and alcohol consumption in both groups were considered, and their differences were not significant.

19 PCs, 8 SMs, 4 LPAs, 3 PAFs, 3 LPCs, 2 Sos, 1 phSM and 1 PE were identified as the discriminating lipid profile of PCV patients by UPLC-MS analysis. Among them, PAF has the AUC ≥0.8, participates in the key pathway of ether lipid metabolism, and involves angiogenesis. It is therefore considered to be the key metabolite in the lipid profile of PCV patients. PAF plays important roles in angiogenesis by increasing the synthesis of various angiogenic factors, such as the vascular endothelial growth factor (VEGF) and matrix metalloproteinase-9^11^,^12^. Meanwhile, PAF-receptor (PAF-R) is present in human RPE cells and choroidal endothelial cells, and can upregulate VEGF in RPE cells^13^. Moreover, PAF-R was detected in a mouse model of laser-induced CNV and was upregulated during CNV development^14^.

PAF is a class of endogenous bioactive phospholipids. There are two different pathways in which PAF can be synthesized: de novo and remodelling pathways. The remodelling pathway is thought to be the primary source of PAF under pathological conditions. The precursor to the remodelling pathway is a phospholipid, which is typically PC. The fatty acid is removed from the sn-2 position of the three-carbon backbone of the phospholipid by phospholipase A2 to produce the intermediate LPC. An acetyl group is then added by LPC acetyltransferase to produce PAF^12^,^13^. As shown in Fig. 2A, the strong correlations (r ≥ 0.5) can be detected between PAFs and other discriminate lipid species, including LPC (22:5), PC(16:0e/19:1), PC(17:1/20:4), PC(20:0p/20:4), PC(16:0e/19:1), PC(17:0/22:6), PC(18:2/18:2), PC(20:4/22:6), SM(d16:0/22:2), SM(d18:1/17:1) and SM(d18:1/19:0). It might provide a hint of the lipid species participating in the PAF metabolism.

PAF is inactivated by the action of PAF-acetylhydrolase (PAF-AH), converting PAF to lysoPAF. PAF-AH is physically associated with lipoproteins in human plasma, primarily low-density lipoprotein (LDL) particles, whereas a small proportion (<20% of total enzyme activity) is associated with HDL. Therefore, HDL and LDL can affect PAF through the association with PAF-AH. As *CETP* polymorphisms is associated with PCV^6^,^7^,^8^,^9^, it would be of interest to further discover the interaction between lipoproteins and PAF in PCV patients.

It should be noticed that the lipidomics analysis with UPLC-MS in this study is comparative and semi-quantitative, since the internal standards were not used. Selection of stable isotopologues as internal standards is necessary for each species to obtain correct quantification. However, the use of numerous different internal standards for each lipid species increases the complexity of samples and in turn hampered to obtain correct quantification^15^. Moreover, it was not our aim to focus on absolute quantification of different species, but to screen potential indicators with this untargeted lipidomics approach, by taking advantage of its breadth of coverage and speed in identifying many compounds. However, validation of the identified indicators is needed and could be performed by quantitative methods such as ELISA.

In summary, the discriminating lipid profile of PCV patients is explored with untargeted UPLC-MS, which contains 41 discriminating lipids. After performing further ROC analysis, pathway analysis and functional analysis, PAF is demonstrated to be the key indicator in the lipid metabolism of PCV patients in our study. Quantification of PAF performed by ELISA demonstrated significantly higher level in PCV patients compared to controls, consistent with the UPLC-MS analysis.

## Methods

### Subjects

The institutional review board at the Zhongshan Ophthalmic Center of Sun Yat-sen University approved a common research protocol for this investigation. The methods were conducted in accordance with the approved guidelines. The content of the informed consent forms was thoroughly discussed with subjects at the time of entry into the study, and verbal and written consent was obtained from all subjects.

A total of 40 subjects were enrolled for the UPLC-MS analysis in this study, including 21 patients with PCV and 19 control individuals. All participants were unrelated Chinese Han individuals who were recruited from the Zhongshan Ophthalmic Center from August 2014 to April 2015. At enrolment, each participant completed detailed lifestyle (including smoking status and alcohol consumption), medical and family history questionnaires. Those with diabetes or CAD were excluded from the study. All PCV patients were newly diagnosed and treatment-naïve. They all underwent bilateral ophthalmic examinations, including visual acuity measurements, slit-lamp biomicroscopy, ophthalmoscopy, colour fundus photography, fluorescein angiography, indocyanine green angiography (ICGA) and optical coherence tomography (Fig. S2). The diagnosis of PCV was based on early subretinal ICGA hyperfluorescence (appearing within the first 5 minute of ICG dye injection) and at least one of the following diagnostic criteria: (1) Nodular appearance of the polyp on stereoscopic viewing; (2) Hypofluorescent halo around the nodule; (3) Abnormal vascular channel(s) supplying the polyps; (4) Pulsatile filling of polyps; (5) Orange subretinal nodules corresponding to the hyperfluorescent area on ICGA; (6) Massive submacular haemorrhage^16^. Participants were excluded if it was difficult to distinguish from nAMD and retinal angiomatous proliferation or if they had other neovascularized maculopathies, such as pathologic myopia, angioid streaks, multifocal choroiditis and punctate inner choroidopathy. All control subjects were matched to PCV participants on gender, age and serum level of TC and TG from cataract department, and they underwent ophthalmic examinations, including visual acuity measurements, slit-lamp biomicroscopy, ophthalmoscopy and 50° colour fundus photography. Those subjects with macular degeneration of any cause, or macular changes (such as drusens or pigment abnormalities) were excluded from the study.

Blood samples were collected following an overnight fast. The samples were left to clot for 30 min and centrifuged. The separated serums were decanted in Eppendorf tubes and stored at −80 °C. TG, TC, HDL-c and LDL-c were evaluated in all subjects.

Differences in the demographic characteristics between PCV patients and controls were assessed using unpaired Student’s t-tests for means, Mann-Whitney tests for medians and chi-squared tests for proportions using GraphPad Prism 7 software (GraphPad Software Inc., La Jolla, CA). A value of p < 0.05 was considered to be statistically significant.

### Lipid profiling using UPLC-MS analysis

#### Lipid extraction

An aliquot of 100 μL of serum was mixed with 600 μL of organic solvent mixture in an Eppendorf tube. The organic solvent mixture consisted of dichloromethane/methanol (2:1, v/v). After intense vortexing for 30 s, samples were centrifuged for 10 min at 12,000 g and 4 °C. The supernatant was dried under a stream of nitrogen at 40 °C and then reconstituted in 200 μL mixture of isopropanol/methanol (1:1, v/v).

#### UPLC–MS analysis

Lipidomic analysis was performed using an Ultimate 3000 (Dionex, USA), which was coupled to a Thermo Orbitrap Elite equipped with a heated electrospray ion source (HESI-II) (Thermo Scientific, USA). The separation of all samples was performed on an Ultimate 3000 Kinetex C18 column (100 × 2.1 mm, 1.9 μm) with the column temperature maintained at 45 °C. The flow rate was 0.4 mL/min, and the mobile phase consisted of acetonitrile–ultrapure water (60:40, v/v) with 10-mM ammonium acetate (A) and isopropanol–ultrapure water (10:90, v/v) with 10-mM ammonium acetate and 0.1% formic acid (B). The gradient conditions for lipid separation were as follows: 0/10, 3/50, 16/99, 18/99, 18.1/10, and 22/10 (min/% solvent B). The sample injection volume was 4 μL. The mass spectrometer was operated in both positive and negative ion modes. The following represent the conditions in positive mode: Heater Temp = 300 °C, Sheath Gas Flow rate = 45 arb, Aux Gas Flow Rate = 15 arb, Sweep Gas Flow Rate = 1 arb, Spray voltage = 3.0 KV, Capillary Temp = 350 °C, S-Lens RF Level = 30%, and Scan range = 200–500. Conditions in negative mode were as follows: Heater Temp = 300 °C, Sheath Gas Flow rate = 45 arb, Aux Gas Flow Rate = 15 arb, Sweep Gas Flow Rate = 1 arb, Spray voltage = 2.5 KV, Capillary Temp = 350 °C, S-Lens RF Level = 60%, and Scan ranges = 200–1500.

Samples were analysed in a randomized order with pooled serum quality control (QC) samples (generated by taking an equal aliquot of all the samples included in the experiment) at regular intervals throughout the run. The overlay of the total ion chromatograms of the QC samples depicted excellent retention time reproducibility (Fig. S3). Internal standards were not included in this untargeted approach.

#### Statistical analysis and validation

First, an unsupervised PCA method was applied to see the natural grouping of samples. PCA is the basis of multivariate modelling and is very useful for outlier detection and for finding patterns and trends. However, instrumental drift, artefacts, and other experimental variation will, on occasion, divert the focus of a PCA model to a systematic variation that is unrelated to the scientific question of interest. Therefore, the resulting principal components do not necessarily align with the best predictive components for class separation, i.e., PCV patients and controls. In such cases, there is a need for a supervised method that makes use of any priori information to refocus the analysis towards the studied objectives, so that the prediction of class membership can be better quantified. Therefore, the supervised OPLS analysis was applied in our study to better identify discriminating lipids that are contributing to the classification of samples and to remove non-correlated variations contained within the spectra. The OPLS score plots and VIP statistics were used for selecting significant variables responsible for group separation. Variables were pre-selected as candidates when their VIP values were larger than 1.0. Then, these features were further subjected to a two-tailed t-test, assuming unequal variance with a threshold of p < 0.05^17^,^18^ and a fold-change comparison. Features were reported as significant and structurally identified if both VIP and t-test p values met the thresholds. When the same metabolite was detected as significant in both MS polarities, the feature with the higher VIP was preferred for further analysis.

The raw UPLC–MS data for all samples were initially processed using Thermo Lipid Search v 4.0.20 Software (Thermo Scientific, USA). The data from each sample were then normalized to total area, and all data were imported into the software SIMCA-P (version 11.5, Umetrics, Umea, Sweden) where multivariate analyses of PCA and OPLS were performed. After the analysis, putative indicators were collected, and their chemical identification was elucidated using the Lipid Search software. Subsequent correlation, clustering heatmap, ROC analysis and pathway analysis were performed by MetaboAnalyst 3.0^19^. Further functional analysis of the metabolites were conducted by searching the public databases as mentioned in the results.

### ELISA

Validation for PAF (Human PAF ELISA Kit; NEO Biolab; Cambridge, MA) was performed by ELISA according to the manufacturer’s manual, with 65 PCV patients (62.43 ± 7.85 years old, 39 male/26 female) and 63 controls (65.35 ± 11.28 years old, 24 male/39 female). In brief, 100 μL of serum or standard was added to the wells in the plate. As the wells have been pre-blocked, no additional blocking steps were applied. 50 μL of Enzyme Solution was then added to each well in the plate and incubate 1 hour at 37 °C. Each well was washed 5 times with 300 μL 1X Wash Solution per well. After the last wash, the plate was inverted and blot dried by tapping on absorbent paper. 50 μL Substrate A was added to each well followed by addition of 50 μL Substrate B, and incubated 15 minutes at room temperature. Then, 50 μL of Stop Solution was added to each well, and immediately read the optical density at 450 nm. All the samples were assayed in duplicate.

## Additional Information

**How to cite this article**: Li, M. *et al*. Analysis of the Serum Lipid Profile in Polypoidal Choroidal Vasculopathy. *Sci. Rep.* **6**, 38342; doi: 10.1038/srep38342 (2016).

**Publisher's note:** Springer Nature remains neutral with regard to jurisdictional claims in published maps and institutional affiliations.

## Supplementary Material

Supplementary Information

## Figures and Tables

**Table 1:** Clinical and demographic characteristics of PCV patients and controls.

Characteristics	Healthy controls (n = 19)	PCV patients (n = 21)	p Value
Age, (mean ± SD), years	64.8 ± 9.2	60.7 ± 9.4	0.174
Male gender (%)	10 (53)	13 (62)	0.554
Body mass index, (Mean ± SD), kg/m^2^	21.8 ± 2.8	22.9 ± 2.5	0.412
History of hypertension (%)	5 (26)	4 (19)	0.865
Smoker (%)	5 (26)	4 (19)	0.865
Alcohol drinker (%)	5 (26)	1 (5)	0.143
Triacylglycerol, [median, (range)], mmol/L	1.7 (1.1, 4.5)	1.5 (0.6, 4.3)	0.057
Total cholesterol (mmol/L)	6.4 (4.6, 8.3)	5.9 (4.8, 8.3)	0.851
HDL-c cholesterol (mmol/L)	5.0 ± 0.8	5.3 ± 0.9	0.282
LDL-c (mmol/L)	4.1 ± 1.0	3.9 ± 1.1	0.499

Statistical analyses were performed using t tests for means, Mann-Whitney tests for medians and chi-square tests for proportions.

PCV, polypoidal choroidal vasculopathy; HDL-c, high-density lipoprotein-cholesterol; LDL-c, low-density lipoprotein-cholesterol.

**Table 2:** List of assigned statistically significant metabolites after comparison of PCV group with the control group.

LipidIon	Ret Time (min)	Calc m/z	VIP	p-Value	Fold Change
UPLC-MS Positive Mode					
Higher in PCV					
LPC(20:4)+H*	1.51	544.3397685	2.464	0.006	0.514
PC(18:0/20:4)+H	26.72	810.6007335	2.447	0.006	0.749
SM(d16:0/22:2)+H*	11.92	757.6218025	2.373	0.008	0.277
PC(16:1p/20:4)+H	9.72	764.5588685	2.185	0.016	3.503
SM(d16:0/26:3)+Na*	12.78	833.6506975	2.140	0.018	0.315
PC(19:0/20:4)+H	12.41	824.6163835	2.113	0.020	0.641
PC(20:3/22:6)+H	10.13	856.5850835	2.091	0.021	0.791
PC(18:1/20:4)+H	10.99	808.5850835	2.066	0.023	0.265
PC(20:1p/19:1)+H	13.03	812.6527685	2.040	0.025	0.521
PC(16:0/22:5)+H	11.48	808.5850835	2.038	0.025	0.765
PC(17:1/20:4)+H	10.41	794.5694335	2.033	0.026	0.811
PC(18:1/24:7)+H	10.60	858.6007335	1.986	0.029	0.689
PC(17:0/20:4)+H	11.32	796.5850835	1.936	0.034	0.532
PC(16:0e/19:1)+H	12.88	760.6214685	1.932	0.034	0.390
LPC(20:1p)+H	3.05	534.3918035	1.921	0.036	0.369
SM(d16:0/20:1)+H*	11.78	731.6061525	1.905	0.037	0.295
SM(d22:1/19:1)+Na*	13.26	821.6506975	1.896	0.038	0.411
SM(d16:0/26:2)+Na	13.73	835.6663475	1.893	0.039	0.312
SM(d16:0/20:2)+H*	10.79	729.5905025	1.821	0.047	0.240
Lower in PCV					
PC(16:0e/22:6)+H	11.71	792.5901685	2.184	0.016	−2.139
So(d16:0)+H	27.82	274.2740555	2.164	0.017	−0.933
So(d22:1)+H	1.89	356.3523055	2.138	0.018	−0.620
PE(18:2/18:2)+H	10.27	740.5224835	2.087	0.022	−0.693
UPLC-MS Negative Mode					
Higher in PCV					
PAF(35:4)+HCOO	11.43	840.5760105	2.169	0.008	0.441
SM(d16:0/22:2)+HCOO*	11.87	801.6127295	2.155	0.009	0.209
LPA(20:4)-H	1.66	457.2360665	2.036	0.014	0.741
SM(d18:1/17:1)+HCOO	10.26	759.5657795	2.004	0.015	0.281
PAF(34:3)+HCOO	11.56	828.5760105	1.933	0.020	0.372
PC(17:0/22:6)+HCOO	11.00	864.5760105	1.891	0.023	0.601
LPC(20:4)+HCOO*	1.56	588.3306955	1.872	0.024	0.326
SM(d16:0/20:1)+HCOO*	11.78	775.5970795	1.823	0.029	0.240
PC(18:2/18:2)+HCOO	10.65	826.5603605	1.801	0.031	0.205
PAF(34:4)+HCOO	10.83	826.5603605	1.794	0.032	0.204
SM(d22:1/19:1)+HCOO*	13.29	843.6596795	1.753	0.036	0.259
phSM(d16:0/26:1)+HCO	13.20	875.6858945	1.750	0.036	0.277
LPC(22:5)+HCOO	1.73	614.3463455	1.727	0.039	0.382
PC(20:4/22:6)+HCOO	9.38	898.5603605	1.703	0.042	0.497
SM(d16:0/20:2)+HCOO*	10.82	773.5814295	1.703	0.042	0.175
SM(d18:1/19:0)+HCOO	12.37	789.6127295	1.699	0.042	0.363
PC(16:0e/22:4)+HCOO	12.34	840.6123955	1.670	0.046	0.307
PC(18:1/20:5)+HCOO	10.19	850.5603605	1.659	0.048	0.367
PC(18:0/20:3)+HCOO	12.46	856.6073105	1.657	0.048	0.723
PC(20:0p/20:4)+HCOO	12.60	866.6280455	1.648	0.049	0.254
SM(d16:0/26:3)+HCOO*	12.91	855.6596795	1.644	0.050	0.232
Lower in PCV					
LPA(18:2)-H	1.82	433.2360665	2.756	0.001	−1.153
LPA(16:0)-H	2.24	409.2360665	2.094	0.011	−0.901
LPA(18:1)-H	2.39	435.2517165	1.823	0.029	−0.905

(*) Detected with statistical significance in both polarities.

TGs, DGs were excluded.
