# Supplementary material for: Analysis of the Serum Lipid Profile in Polypoidal Choroidal Vasculopathy
Source: Sci Rep. 2016 Dec 2;6:38342. doi: 10.1038/srep38342 (PMC5133601; doi:10.1038/srep38342)
Supplement: Supplementary Information [file srep38342-s1.pdf]

Analysis of the Serum Lipid Profile in Polypoidal Choroidal Vasculopathy

Miaoling Li; Xiongze Zhang; Nanying Liao; Baikang Ye; Yuting Peng; Yuying Ji; Feng Wen*

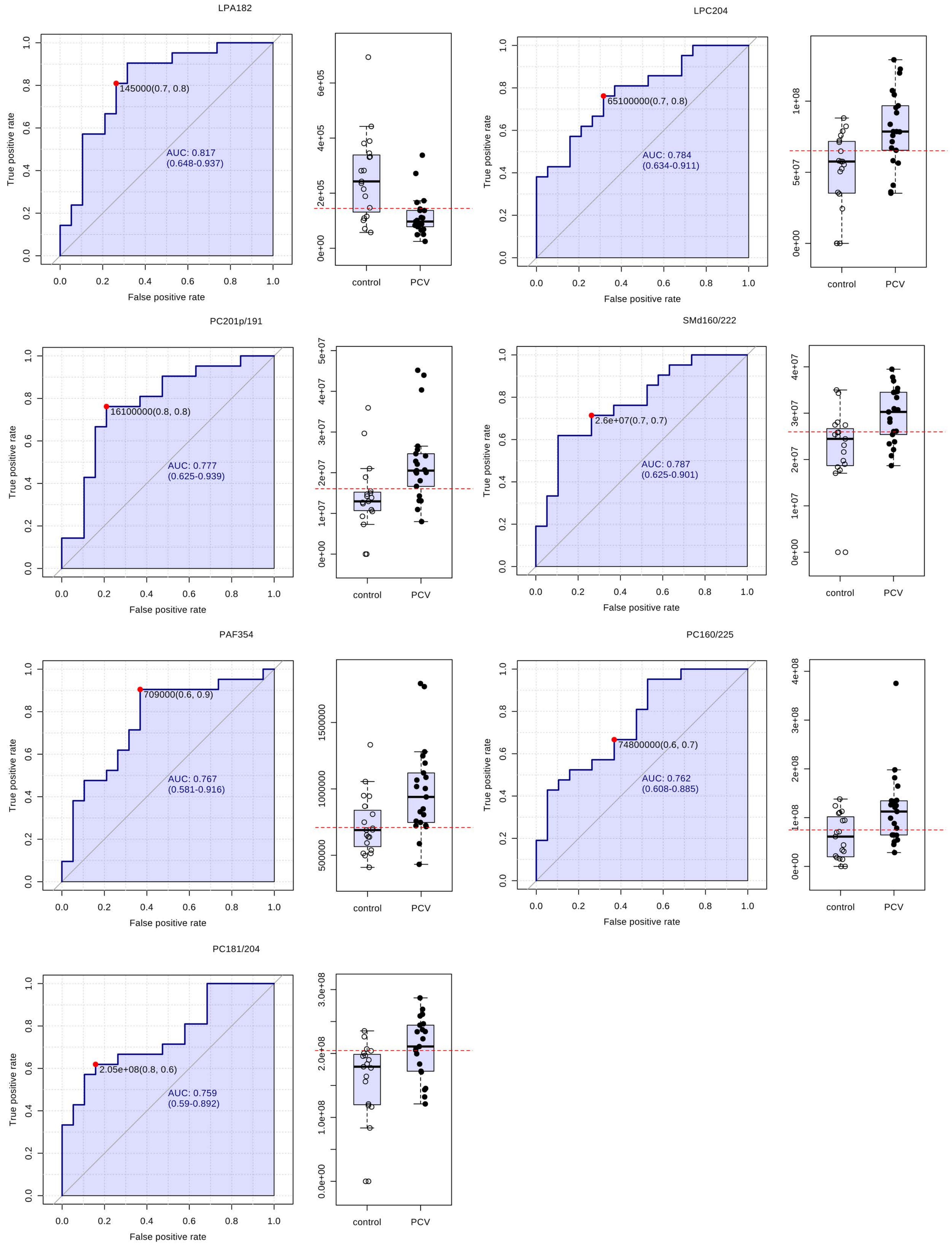

LPA182
145000(0.7, 0.8)
AUC: 0.817
(0.648-0.937)
True positive rate
False positive rate
control
PCV
LPC204
65100000(0.7, 0.8)
AUC: 0.784
(0.634-0.911)
True positive rate
False positive rate
control
PCV
PC201p/191
16100000(0.8, 0.8)
AUC: 0.777
(0.625-0.939)
True positive rate
False positive rate
control
PCV
SMd160/222
2.6e+07(0.7, 0.7)
AUC: 0.787
(0.625-0.901)
True positive rate
False positive rate
control
PCV
PAF354
709000(0.6, 0.9)
AUC: 0.767
(0.581-0.916)
True positive rate
False positive rate
control
PCV
PC160/225
74800000(0.6, 0.7)
AUC: 0.762
(0.608-0.885)
True positive rate
False positive rate
control
PCV
PC181/204
2.05e+08(0.8, 0.6)
AUC: 0.759
(0.59-0.892)
True positive rate
False positive rate
control
PCV

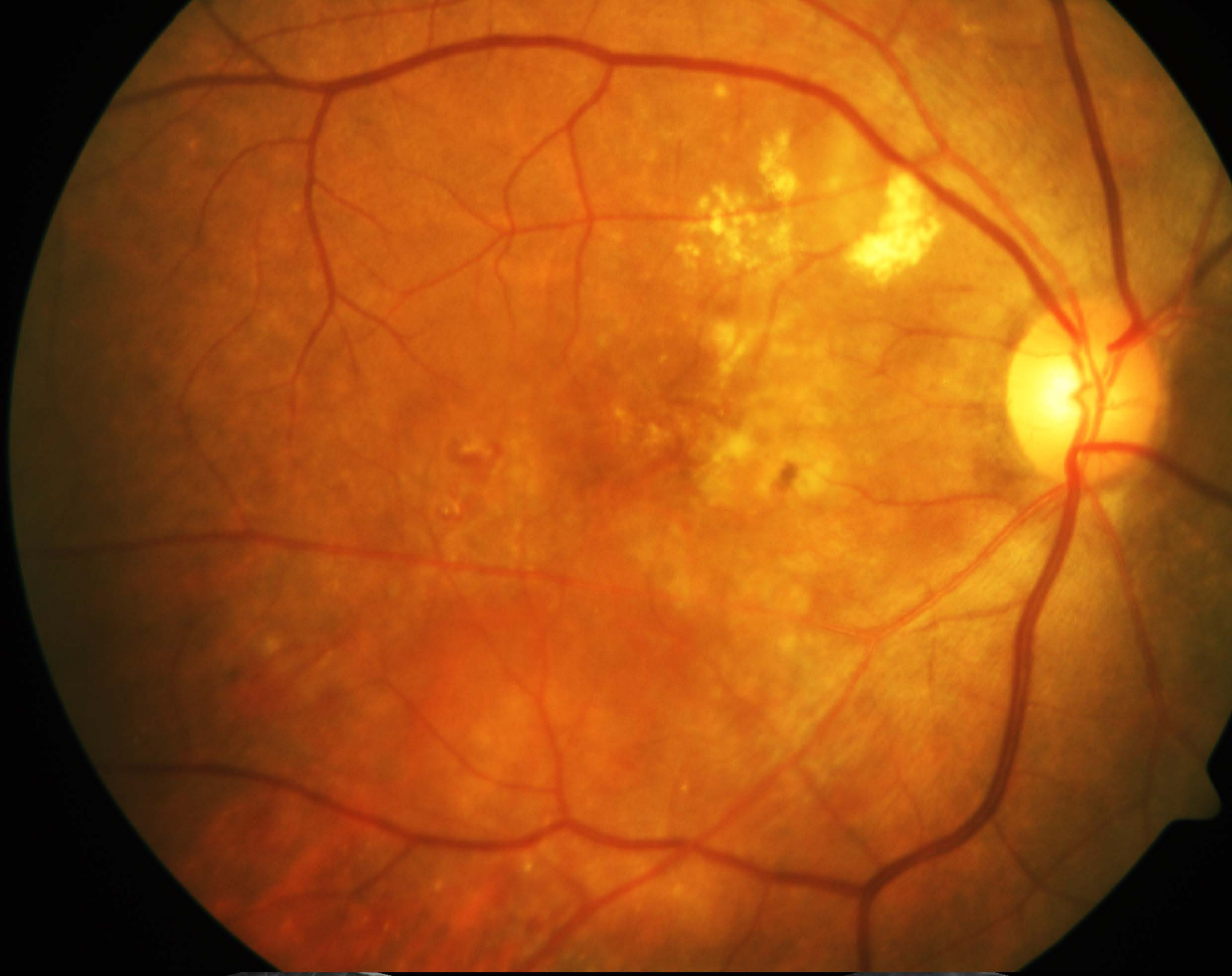

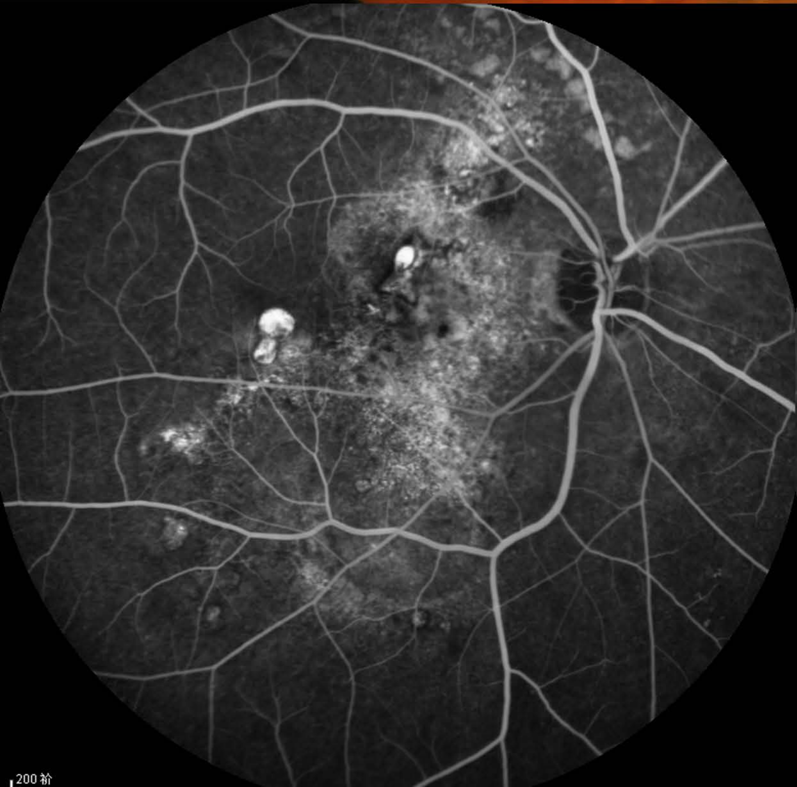
200 祄

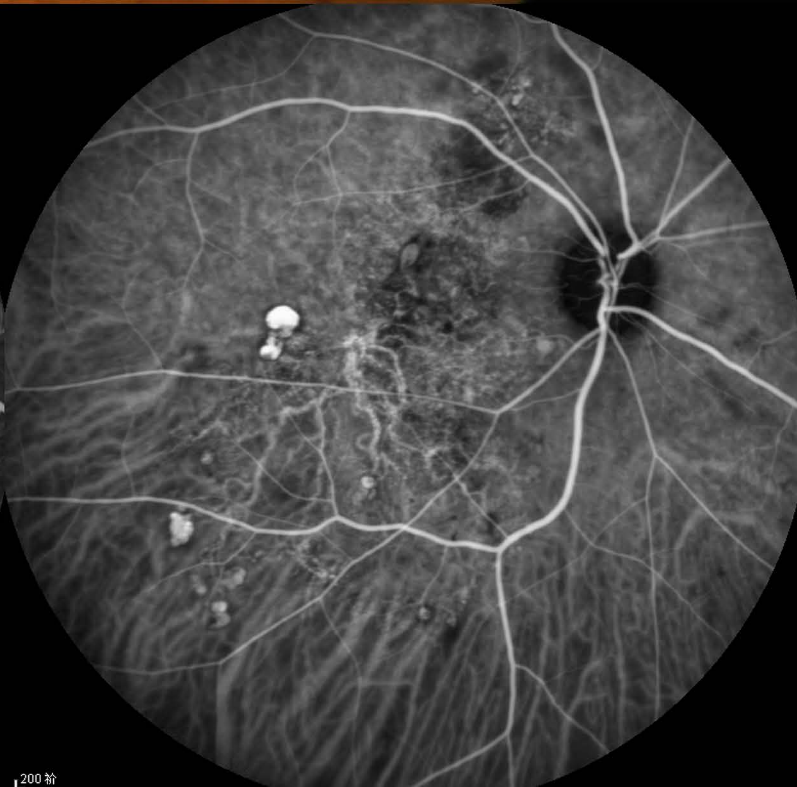
200 祄

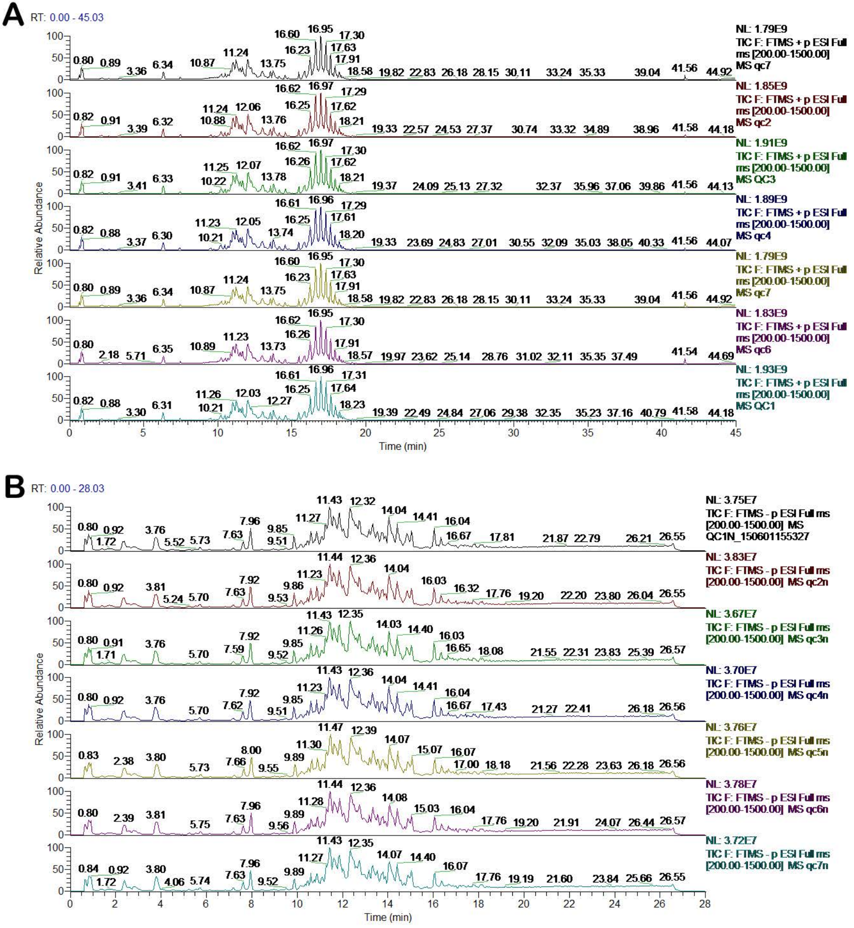
A
RT: 0.00 - 45.03
NL: 1.79E9 TIC F: FTMS + p ESI Full ms [200.00-1500.00] MS qc7
NL: 1.85E9 TIC F: FTMS + p ESI Full ms [200.00-1500.00] MS qc2
NL: 1.91E9 TIC F: FTMS + p ESI Full ms [200.00-1500.00] MS QC3
NL: 1.89E9 TIC F: FTMS + p ESI Full ms [200.00-1500.00] MS qc4
NL: 1.79E9 TIC F: FTMS + p ESI Full ms [200.00-1500.00] MS qc7
NL: 1.83E9 TIC F: FTMS + p ESI Full ms [200.00-1500.00] MS qc6
NL: 1.93E9 TIC F: FTMS + p ESI Full ms [200.00-1500.00] MS QC1
Relative Abundance
Time (min)
B
RT: 0.00 - 28.03
NL: 3.75E7 TIC F: FTMS - p ESI Full ms [200.00-1500.00] MS QC1N_150601155327
NL: 3.83E7 TIC F: FTMS - p ESI Full ms [200.00-1500.00] MS qc2n
NL: 3.67E7 TIC F: FTMS - p ESI Full ms [200.00-1500.00] MS qc3n
NL: 3.70E7 TIC F: FTMS - p ESI Full ms [200.00-1500.00] MS qc4n
NL: 3.76E7 TIC F: FTMS - p ESI Full ms [200.00-1500.00] MS qc5n
NL: 3.78E7 TIC F: FTMS - p ESI Full ms [200.00-1500.00] MS qc6n
NL: 3.72E7 TIC F: FTMS - p ESI Full ms [200.00-1500.00] MS qc7n
Relative Abundance
Time (min)

Fig. S1. Receiver Operating Characteristic (ROC) curves of the lipid species with area under the ROC curve (AUC) ⩾0.8. The associated AUC, 95% CI, sensitivities, and specificities are indicated.

Fig. S2. Representative colour fundus photography, fluorescein angiography, and indocyanine green angiography of the PCV patients.

Fig. S3. Total ion chromatogram of quality control samples in positive mode (A) and negative mode (B), demonstrating excellent retention time reproducibility.
